# COVID-19 and public support for autonomous technologies—Did the pandemic catalyze a world of robots?

**DOI:** 10.1371/journal.pone.0273941

**Published:** 2022-09-28

**Authors:** Michael C. Horowitz, Lauren Kahn, Julia Macdonald, Jacquelyn Schneider

**Affiliations:** 1 Department of Political Science, University of Pennsylvania, Philadelphia, Pennsylvania, United States of America; 2 Council on Foreign Relations, Washington, D.C., United States of America; 3 Department of Political Science, University of Denver, Denver, Colorado, United States of America; 4 Freeman Spogli Institute, Stanford University, Stanford, California, United States of America; Universitat Luzern, SWITZERLAND

## Abstract

By introducing a novel risk to human interaction, COVID-19 may have galvanized interest in uses of artificial intelligence (AI). But was the pandemic a large enough catalyst to change public attitudes about the costs and benefits of autonomous systems whose operations increasingly rely on AI? To answer this question, we use a preregistered research design that exploits variation across the 2018 and 2020 waves of the CCES/CES, a nationally representative survey of adults in the United States. We compare support for autonomous cars, autonomous surgeries, weapons, and cyber defense pre- and post-the beginning of the COVID-19 pandemic. We find that, despite the incentives created by COVID-19, the pandemic did not increase support for most of these technologies, except in the case of autonomous surgery among those who know someone who died of COVID-19. The results hold even when controlling for a variety of relevant political and demographic factors. The pandemic did little to push potential autonomous vehicle users to support adoption. Further, American concerns about autonomous weapons, including cyber defense, remain sticky and perhaps exacerbated over the last two years. These findings suggest that the relationship between the COVID-19 pandemic and the adoption of many of these systems is far more nuanced and complex than headlines may suggest.

## Introduction

In early 2020, an unseen and microscopic biological threat spread across the globe. While the world struggled to combat the SARS-CoV-2 virus, economies, societies, and public health institutions turned to digital networks and technologies to make physical isolation possible. New robotic innovations, designed to keep humans safe and healthy, debuted as a response to the pandemic. From automated food delivery to avatar bedside doctors, robotic sentries, and temperature takers—COVID-19 catalyzed a global interest in artificial intelligence (AI) and autonomous technologies [1–3].

The COVID-19 pandemic likely accelerated trends toward automation in the US economy [4, 5]. For example, one survey suggested that 68% of US businesses increased investments in automation during the pandemic [6]. However, public support and confidence is key to technology adoption [7–10]. So have public attitudes shifted along with business investment? Studying the public’s embrace or aversion is crucial because many questions about autonomous systems, for example around the use of self-driving cars, are questions at the intersection of politics and psychology [11, 12].

More generally, understanding public attitudes about technology adoption is essential for multiple reasons. First, public attitudes represent microfoundations for how those drivers end up impacting public policy, especially when attitudes are polarized [13–19]. Second, public attitudes can influence how elites view important policy issues surrounding AI-enabled autonomous systems [20–22], thus influencing policymaking. Third, for salient AI-enabled autonomous systems such as autonomous vehicles, public acceptance will determine whether the technology succeeds in the marketplace [23–27]. Finally, elite and general public attitudes on most public policy topics are not as different as many assume, meaning measuring public attitudes can also generate insights into elite perspectives [28].

To understand how COVID-19 impacts support for AI-enabled autonomous systems, we use a pre-registered design (on Open Science at 10.17605/OSF.IO/RVC9S) to compare support for four types of AI-enabled autonomous systems—autonomous vehicles, autonomous surgery, autonomous cyber defense, and autonomous weapon systems—between two identical surveys fielded on a representative sample of 1,000 US adults in fall 2018 and fall 2020. The passage of time across surveys taken prior to and after the beginning of the COVID-19 pandemic can help us evaluate the role of COVID-19 in affecting public preferences around these contentious technologies.

The results suggest that, despite the incentives created by COVID-19, and growing corporate investments in automation, there was not a straight correlation between the pandemic and increased public support for many autonomous technologies. Instead, we found slightly declining support across most issue areas, except in the case of autonomous surgery (and even then, only among those who know someone who died of COVID-19). Even though the survey was fielded at the height of concern about the contagion and lock-down, we also find little push for potential autonomous vehicle users to support adoption. Finally, American concerns about autonomous weapon systems and cyber defense remain sticky.

## Theory and hypotheses

How might COVID-19 impact support for the adoption of AI-enabled autonomous systems? We define artificial intelligence as the capability for machines to conduct tasks once thought to require human intelligence. Artificial intelligence methods like machine learning are one way to program autonomous systems, systems that operate with minimal or no human oversight [29]. Perhaps the logical hypothesis given the concern in 2020 about close contact and the general increase in fear of human interaction and disease transmission, is that the pandemic would increase support for uses of AI-enabled autonomous systems, especially systems that limit contact with other humans. However, this is also a relatively short period between the 2018 and 2020 surveys without clear technological breakthroughs. Therefore, conversely, COVID-19 might not provide a galvanizing effect to change public views about autonomy.

While we don’t purport to generalize about how COVID-19 may affect the adoption of all autonomous technologies, we are interested in technologies that vary in three ways: 1) civilian vs. military applications, 2) public salience or awareness of technologies, and 3) technological maturity. This led us to choose four technology baskets: autonomous vehicles, autonomous surgery, autonomous weapon systems, and autonomous cyber defense.

Our two civilian applications, autonomous vehicles and autonomous surgery, vary in public salience but have similarities in technological maturity. Autonomous vehicles, for example, often feature prominently in public discussions, and levels of autonomy (for example self-parking and even some self-driving applications) are becoming increasingly common among the general public [30]. This means that there is high public salience or awareness of autonomous vehicles. However, high awareness does not necessarily mean high adoption. Studies on the adoption of autonomous vehicles show that perceptions of these technologies vary across populations [31–34], often driven by beliefs about the maturity of the technology [35]. Similarly, beliefs about the technological maturity of autonomous surgery also vary across populations. Like cars, fully autonomous surgery is not mainstream; however, robot-aided surgery and uses of autonomy in surgery are increasingly commonplace [36, 37]. Unlike autonomous vehicles, however, the debate about autonomous surgery is largely among experts [38]. In fact, in a recent survey of the British public, researchers found that over 80% of respondents mistakenly believed that fully autonomous surgeries already occurred [39]. These two cases, therefore, show similarities in technological maturity and their civilian uses but differ in their public knowledge and salience.

Like our two civilian applications, lethal autonomous weapons and automated cyber defenses, vary in public salience but are largely similar in terms of technological maturity and neither features significant public knowledge. Lethal autonomous weapons are perhaps some of the most emotionally salient applications of the technology and surveys routinely find strong distaste for these systems within the public (far exceeding our other civilian and military cases) [21, 40]. They are also increasingly technologically available, with examples of autonomy ranging from AI-enabled targeting to missile seekerheads and even fully autonomous loitering munitions and mines [41]. Autonomous cyber defenses are perhaps the outlier in autonomous weapons systems. First, they are a defensive technology—which decreases public discussion. Secondly, cyber operations are typically viewed differently than other weapon systems [42, 43], which means that their salience is much lower than other kinetic weapon systems. While these technologies may not be high in public salience or awareness, their technological maturity is probably the most advance of the technologies we examined with examples of functioning cyber defense autonomy as early as DARPA’s Grand Challenge in 2016 [44].

Finally, (and perhaps most importantly for this paper) while not an explicit variable that we selected cases on in 2018, our technologies of interest also feature variance in their COVID risk transference, with autonomous surgery and cars offering a risk mitigation option for elements of the general population while autonomous weapons decrease COVID-19 risk for manned military operations and (in contrast) cyber defenses have little effect on COVID-19 risk transference.

### Civilian applications: Autonomous vehicles and autonomous surgery

If COVID-19 led to more support for AI-enabled autonomous systems, the period between fall 2018 and fall 2020 should see an increase in support for civilian uses of AI-enabled autonomy—such as self-driving cars and autonomous surgery. First, the COVID-19 pandemic could make people more comfortable with AI-enabled autonomous systems because of transference of risk [45–47]. Before COVID-19, users may have seen the primary risk of adoption as the risk of delegating dangerous decision-making to the machine. However, during COVID-19, users may have instead viewed the primary risk as that of contracting COVID-19 from human contact. Transportation options that would not involve contact with other humans, such as autonomous taxis, should become more attractive. Similarly, the need for medical care and surgery still exists despite the COVID-19 pandemic. Thus, people should become more supportive of autonomous surgery because it enables needed medical procedures without the risk of COVID-19 transmission from human contact. After all, COVID-19 made many Americans more likely to delay or avoid in-person medical care to reduce the risk of contracting COVID-19, instead opting for telehealth visits or no health care at all [48, 49]. At the same time, hospitals sought out robotic and autonomous options for sanitizing, routine patient interaction, and even triage with COVID-infected patients—making the health sector the most likely context in which people may have encountered novel autonomous adoption [50, 51]. Second, if the increased use of AI between 2018 and 2020 (some tied to the pandemic and others not) also led to an increase in self-reported personal use, this could lead to higher levels of support due to increased familiarity with AI technologies [52]. We would also expect technological improvements to drive an increase in approval of AI.

*Hypothesis 1*: *Support for autonomous vehicles and surgery will be higher in fall 2020 than in fall 2018*.

Alternatively, it is possible that, despite COVID-19, opinions about AI-enabled autonomous systems remain unchanged. For example, while there may have been an increased appetite for autonomy in many health venues, there was not a concurrent breakthrough in AI technological capabilities (especially one salient to the general public). Further, public attitudes about autonomous systems in high physical risk situations could be too sticky to be affected by COVID-19. Research suggests a general human aversion to using algorithms in high-pressure situations, especially when there is a risk of accidents [53]. To the extent that autonomous vehicles have made headlines in the last few years, it has been through lethal accidents [54]. Moreover, increasing discomfort with and worry over healthcare due to higher levels of awareness of COVID-19 risks could be expressed as a lack of support for any in-person healthcare, including autonomous surgery. As a result, we also hypothesize that:

*Hypothesis 2*: *Support for autonomous vehicles or autonomous surgery should not be higher in the relevant 2020 CCES questions compared to the 2018 CCES*.

While autonomous vehicles and surgery may at first seem similarly impacted by COVID-19, COVID-19 might have created unique dynamics for autonomous surgery that did not exist pre-pandemic. As reasoned above, individuals might view autonomous surgery as a way to insulate both themselves and healthcare workers from the risk of COVID-19. In particular, this should be the case among those with direct experience with the hospital system during the COVID-19 pandemic. This group should be more primed to recognize the potential benefits presented above, making them relatively more supportive of autonomous surgery.

*Hypothesis 3*: *Support for autonomous surgery should decrease between the 2020 and 2018 CCES, except among those most negatively impacted by COVID-19 in ways that directly involved the medical system, meaning knowing someone who was hospitalized and died of COVID-19*.

### Military applications: Autonomous cyber defense and autonomous weapons

Shifting to the effect of COVID-19 on support for military uses of AI-enabled autonomous systems, COVID-19 might increase support for AI-enabled weapons, because AI-enabled weapons are associated with uninhabited platforms which may lower the risk of US military personnel contracting COVID-19 on missions. In particular, the highly salient COVID-19 outbreak on the USS Theodore Roosevelt in the spring of 2020, coupled with a public discussion about COVID-19 impact on US military forces throughout the globe could make the public more likely to support autonomous weapon systems that allow US personnel to fight from a safe distance (from each other and COVID-19-risky deployments) [55]. Related, as the world became more dependent on digital technologies during the pandemic [56], individuals might also be more risk acceptant of cyber operations that keep those digital capabilities secure.

*Hypothesis 4*: *COVID-19 increases support for AI-enabled weapons and cyber operations*.

Our previous discussion presumes an informed public that understands the risks of COVID-19 to military readiness and digital capabilities. However, the public is not always well informed about these subjects and may have limited knowledge of the military readiness issues created by COVID-19 or the details of military capabilities overall, meaning they may not connect military operations with the risk of COVID-19 transmission. Moreover, the cyber realm could already seem independent of COVID-19, so the pandemic would not influence attitudes about AI-enabled autonomous cyber defense. For these reasons, the US public’s attitudes towards AI-enabled autonomous weapon systems and cyber defense may be unaffected by the pandemic.

*Hypothesis 5*: *COVID-19 has no effect on public support for AI-enabled weapons or cyber operations*.

## Methodology

We test these hypotheses about the relationship between COVID-19 and popular support for AI-enabled autonomous systems by exploiting variation between the 2018 and 2020 waves of the Cooperative Congressional Election Study (CCES), now called the Cooperative Election Study (CES) [57, 58]. The 2018 survey was fielded on 1,000 individuals in two phases—before and after the November 2018 general elections in the United States, and the 2020 survey was also fielded on 1,000 individuals in two phases—before and after the November 2020 general elections in the United States. Both samples were representative samples of US adults [57, 58]. A module in the 2018 CCES featured questions about attitudes surrounding the adoption of AI-enabled autonomous systems across the technologies described above. We then included the same questions in the 2020 CES, meaning we can exploit the natural experiment of the time difference between October 2018 and October 2020 to test our hypotheses. The study was preregistered using Open Science at 10.17605/OSF.IO/RVC9S.

The study was judged exempt from Human Subjects Review under the University of Pennsylvania IRB Protocol 828933. Written consent was obtained by the online survey firm YouGov for all participants. All participants were U.S. adults.

There was not a publicly salient change in publicly available AI-enabled autonomous systems between 2018–2020. Stories about crashes of autonomous vehicles existed both before and after 2018, making crashes during the period unlikely to shift attitudes. Stories that might influence attitudes about technology companies are unlikely to influence attitudes about particular AI-enabled autonomous systems, especially as unpopular technology companies such as Facebook are not major producers of any of the technologies examined in this paper. We can further control for the impact of demographic factors and partisanship in regression models, as shown in S1 and S2 Tables.

The dependent variables are whether respondents support the adoption of autonomous vehicles, autonomous surgery, autonomous cyber defense, and autonomous weapon systems. Each support question contained a four-point scale, where 1 represents *very unsupportive* and 4 represents *very supportive*. Full details on the coding of each item are available in S1 File. Tables 1 and 2 show the distribution of our key demographic variables across the 2018 and 2020 surveys. We detail how we operationalize key independent and control variables below.

- Sex (1 if female, 0 if male)
- Age (Count)
- Military Service (1 if yes, 0 otherwise)
- Level of education (1–6, where 1 = did not complete high school and 6 = graduate degree)
- Political party (1–7, where 1 = strong Democrat and 7 = strong Republican)
- Use of Ridesharing Apps (1 if respondent has used ridesharing apps before the COVID-19 pandemic, 0 otherwise. Asked in 2020 only.)
- Drive (1 if respondent has a driver’s license and 0 otherwise. Asked in 2020 only.)
- Urbanization (1–4, where 1 = living in a city and 4 = living in a rural area)
- Respondent lives in top 10 Auto Manufacturing State (Vehicles model only. Data from [59].)
- Respondent lives in top 10 Health Care Employment State (Surgery model only. Data from [60].)
- Self-reported level of prior experience with AI (0–5 scale where 0 is lowest and 5 is highest)
- COVID-19 Death (1 if family or friend died of COVID-19, 0 otherwise)

**Table 1:** Summary statistics CCES 2018.

Variable	N	Mean	Median	St. Dev.	Min	Max
Sex	1000	0.578	1	0.494	0	1
Age	1000	49.026	50	17.752	19	96
Education Level	1000	3.632	3	1.543	1	6
Family Income	901	6.284	6.000	3.341	1.000	16.000
Political Party	960	3.723	4.000	2.239	1.000	7.000
Urbanization	992	2.240	2.000	1.058	1.000	4.000
Auto Manufacturing State	1000	0.183	0	0.387	0	1
Hospital Employment	1000	0.079	0	0.270	0	1
AI Experience	1000	0.470	0	0.705	0	2

**Table 2:** Summary statistics CES 2020.

Variable	N	Mean	Median	St. Dev.	Min	Max
Sex	1000	0.562	1	0.496	0	1
Age	1000	49.293	50	17.637	19	89
Education Level	1000	3.611	3	1.487	1	6
Family Income	913	6.323	6.000	3.453	1.000	16.000
Political Party	951	3.481	3.000	2.176	1.000	7.000
Use of Ridesharing Apps	933	0.395	0.000	0.489	0.000	1.000
Drive	1000	0.874	1	0.332	0	1
Urbanization	995	2.198	2.000	1.048	1.000	4.000
Auto Manufacturing State	1000	0.166	0	0.372	0	1
Hospital Employment	1000	0.079	0	0.270	0	1
AI Experience	1000	1.256	1	1.131	0	5

S1 and S2 Figs detail the correlations between key independent variables, including those outlined above. The summary statistics for our variables of interest are below in Tables 1 and 2.

In the results that follow, we begin with comparisons of means of levels of support for each technology between 2018 and 2020. We then use regression models to control for demographic, political, and other confounders that could influence those levels of support. Given the continuous character of the dependent variables, we employ ordinary least squares (OLS) models when using regression analysis. Because the surveys were not a panel design, meaning different respondents received the survey in 2018 and 2020, we estimate independent regression models for 2018 and 2020, rather than pooling all of the data. The results below are substantively and statistically identical when ordered logit models for the dependent variables or when using team weights designed to make the sample more representative of US adults or not using team weights.

## Results

**Fig 1:**
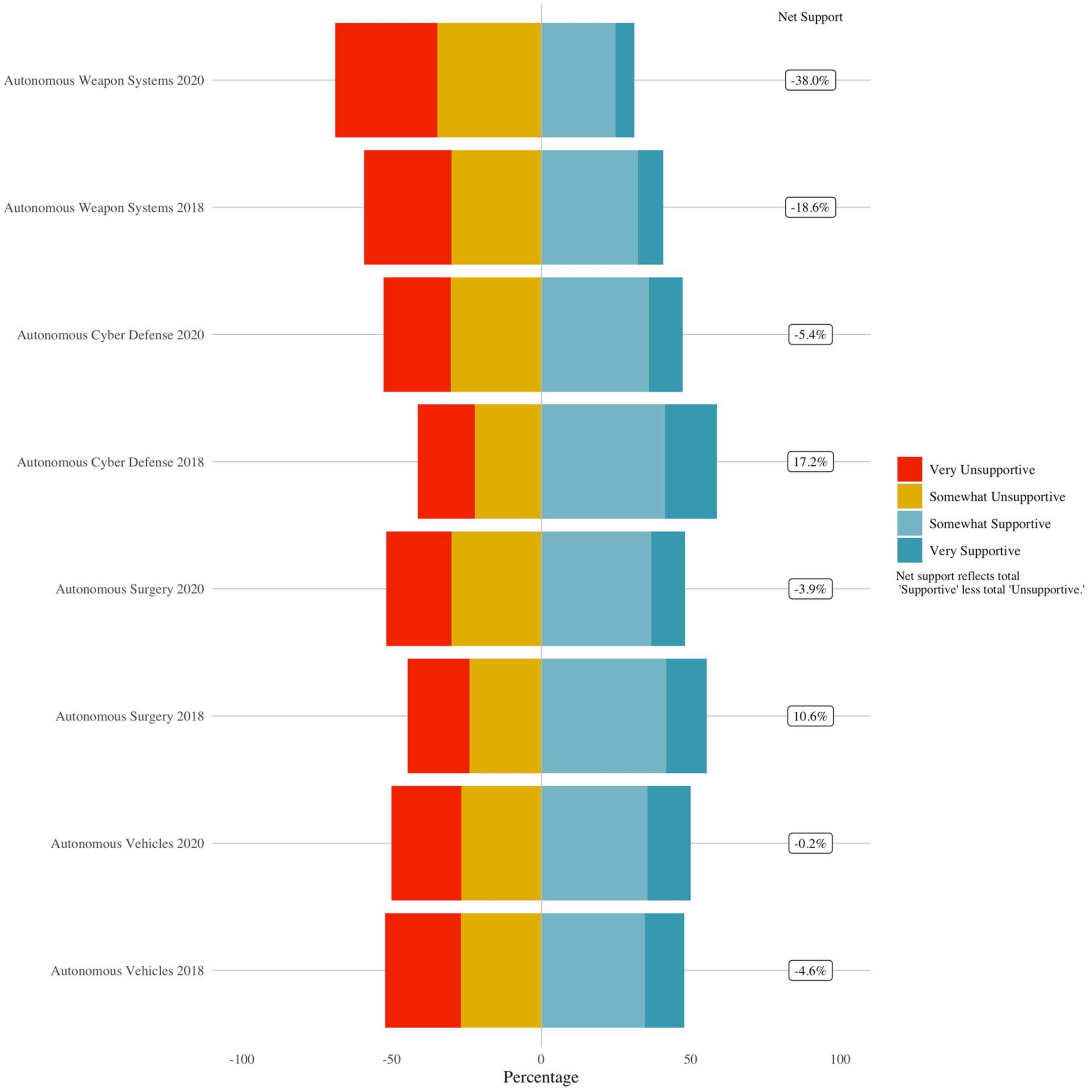
Change in support for AI-enabled autonomous systems from 2018 to 2020.

We proceed by evaluating perceptions of civilian applications of AI, vehicles and surgery, and then military applications of AI, cyber defense and weapon systems. As described above in the methodology section, we start by looking at means and standard errors for each year for each type of AI-enabled autonomous system. Fig 1 below illustrates the level in 2018 and 2020 for each AI-enabled autonomous system. Those somewhat or very supportive of self-driving cars increased from 47% to 50% between 2018 and 2020. Support for all other technologies declined, some substantially. For example, support for automated cyber defenses decreased from 59% to 48% from 2018 to 2020.

### Autonomous vehicles and autonomous surgery

**Fig 2:**
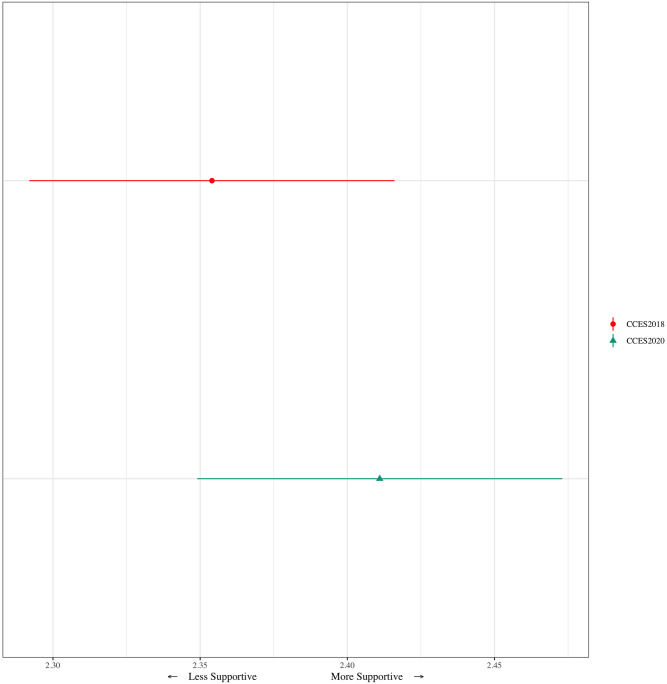
Support for autonomous vehicles, 2018 vs. 2020.

We start by testing hypotheses 1 and 2, on the likely relationship between COVID-19 and support for autonomous vehicles. We compare the support mean for autonomous vehicles in 2018 to the support mean in 2020 in Fig 2. Given the lack of exogenous, salient changes in autonomous vehicle technology between 2018 and 2020, an increase in support would suggest fear of human contact due to COVID-19 made respondents more willing to use autonomous vehicles. The results, however, support hypothesis 2. Despite COVID-19 leading to fear of human contact, that fear did not lead to an increase in support for autonomous vehicles. Average support for autonomous vehicles slightly increased from 2018 (2.35) to 2020 (2.41), but the difference is not statistically significant. In 2018, 47.7% were somewhat or very supportive, increasing to 49.9% in 2020.

We turn to regression analysis using OLS to unpack the key drivers of support for autonomous vehicles. The dependent variable is support for autonomous vehicles, and the independent variables are the list of independent variables described in the methodology section. Fig 4 shows the coefficient values and 95% confidence intervals for the regression output, which we can interpret as substantive effects given the OLS model specification. The regression table is available in S1 and S2 Tables.

The results show consistency in the drivers of attitudes about autonomous vehicles across the two periods, with one exception. In both years, self-reported prior experience with AI is associated with substantially greater support for autonomous vehicles. Women and older respondents are statistically significantly (*p* < 0.05) less supportive of autonomous vehicles. More educated respondents are more supportive of autonomous vehicles, but these effects are not consistently statistically significant (*p* < 0.05)). Interestingly, being in a top 10 auto manufacturing state made support for autonomous vehicles significantly more likely in 2018, but significantly less likely in 2020. Though the sample size is too small to allow meaningful analysis, we can speculate that, in combination with the COVID-19 pandemic, respondents may have feared job loss from automation more in 2020 than in 2018 in the auto industry. In 2018, they may have viewed the production of autonomous vehicles as a potential positive for their states. Partisanship also cannot fully explain these results. In 2018, there was no significant difference between support for autonomous vehicles between self-identified Democrats and Republicans. In 2020, Democrats were significantly more supportive, with a shift from being a strong Republican to a strong Democrat creating a 7% increase in the probability of support for autonomous vehicles. One explanation for these results is the shift of college-educated voters towards the Democratic party during the Trump years. Given that more educated respondents are somewhat more likely to support autonomous vehicles, that could explain the shift, though it was not part of the pre-registered design.

Turning to autonomous surgery, we begin again by looking at mean levels of support. Support for autonomous surgery declined from 2018 (2.48) to 2020 (2.37), and the difference is statistically significant at the 0.05 level. Those that were somewhat or very supportive declined from 45.3% in 2018 to 38.05% in 2020. Again, in the absence of publicly salient technological changes, it seems plausible to attribute at least some of this difference to COVID-19, especially since COVID-19 may have led to concern about using the health care system. We also find support for the second part of hypothesis 3, that those with direct experience with the hospital system during the COVID-19 pandemic, defined based on a variable in the 2020 CES as those that personally knew people that died of COVID-19, should be more supportive of autonomous surgery. Given that most COVID-19 deaths involved hospitalization prior to death, this variable is a reasonable proxy for awareness of the interaction between COVID-19 and health care risks.

**Fig 3:**
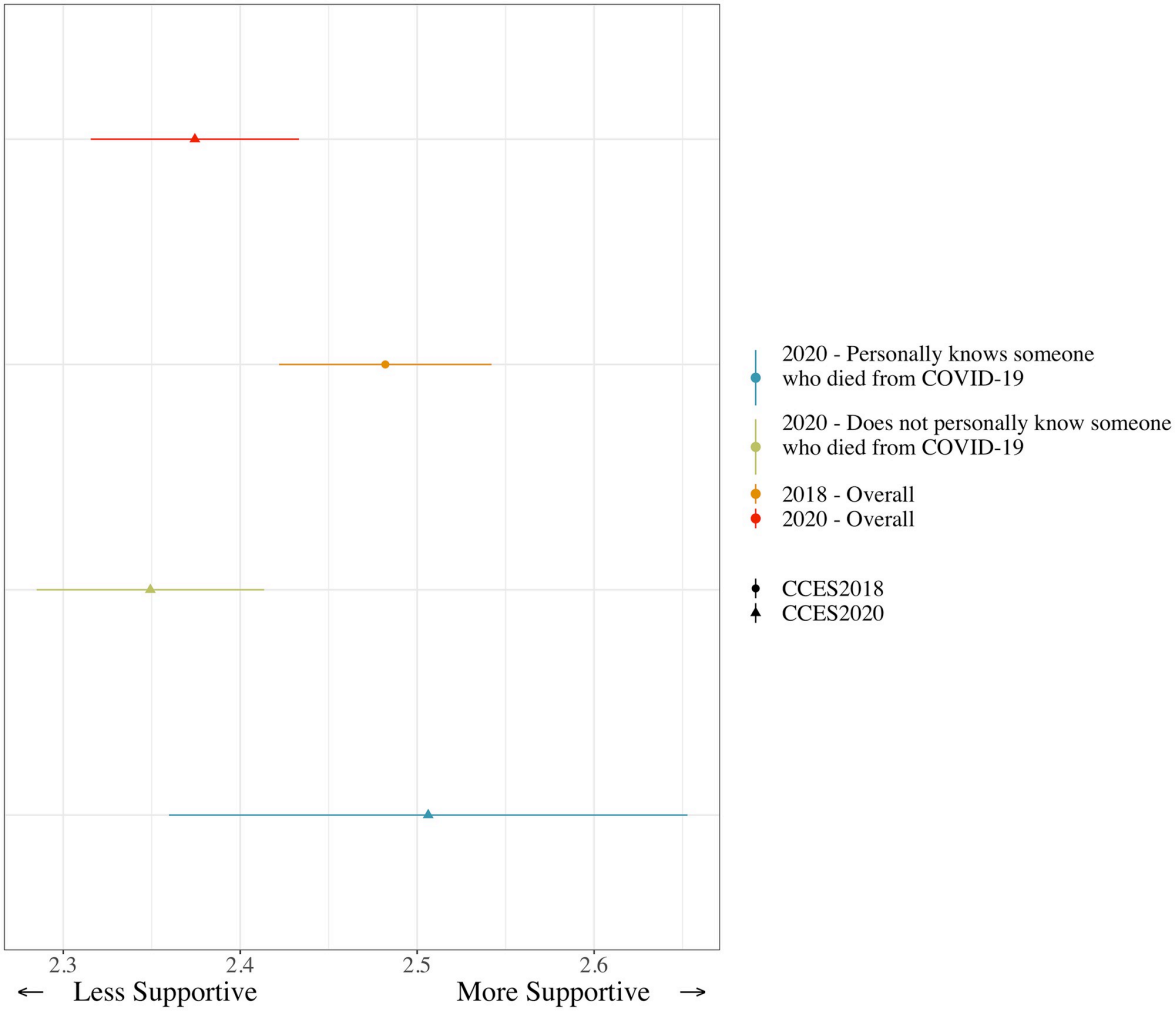
Support for autonomous surgery, 2018 vs. 2020, and among those who experienced a personal loss due to COVID-19.

As Fig 3 shows, 2020 CES respondents that knew people who died of COVID-19 were significantly more supportive of autonomous surgery (average support score of 2.51) than those who did not know people who died of COVID-19 (average support score of 2.35). The difference is statistically significant at the 0.05 level, and 2018 CCES respondents, overall, were also significantly more supportive, at the 0.01 level, of autonomous surgery than 2020 respondents who did not know someone who died of COVID-19. This suggests potentially the desire to protect frontline health care workers (or self) may influence support for autonomous surgery. It is also important to note that the 2020 survey was conducted at the height of the initial wave of COVID-19 in which there were fewer individuals with direct relationships with someone who had died of COVID-19 than in later years. These experiences therefore could have been extremely evocative and important for these individuals’ perceptions of healthcare and the risk from treatment during the pandemic.

**Fig 4:**
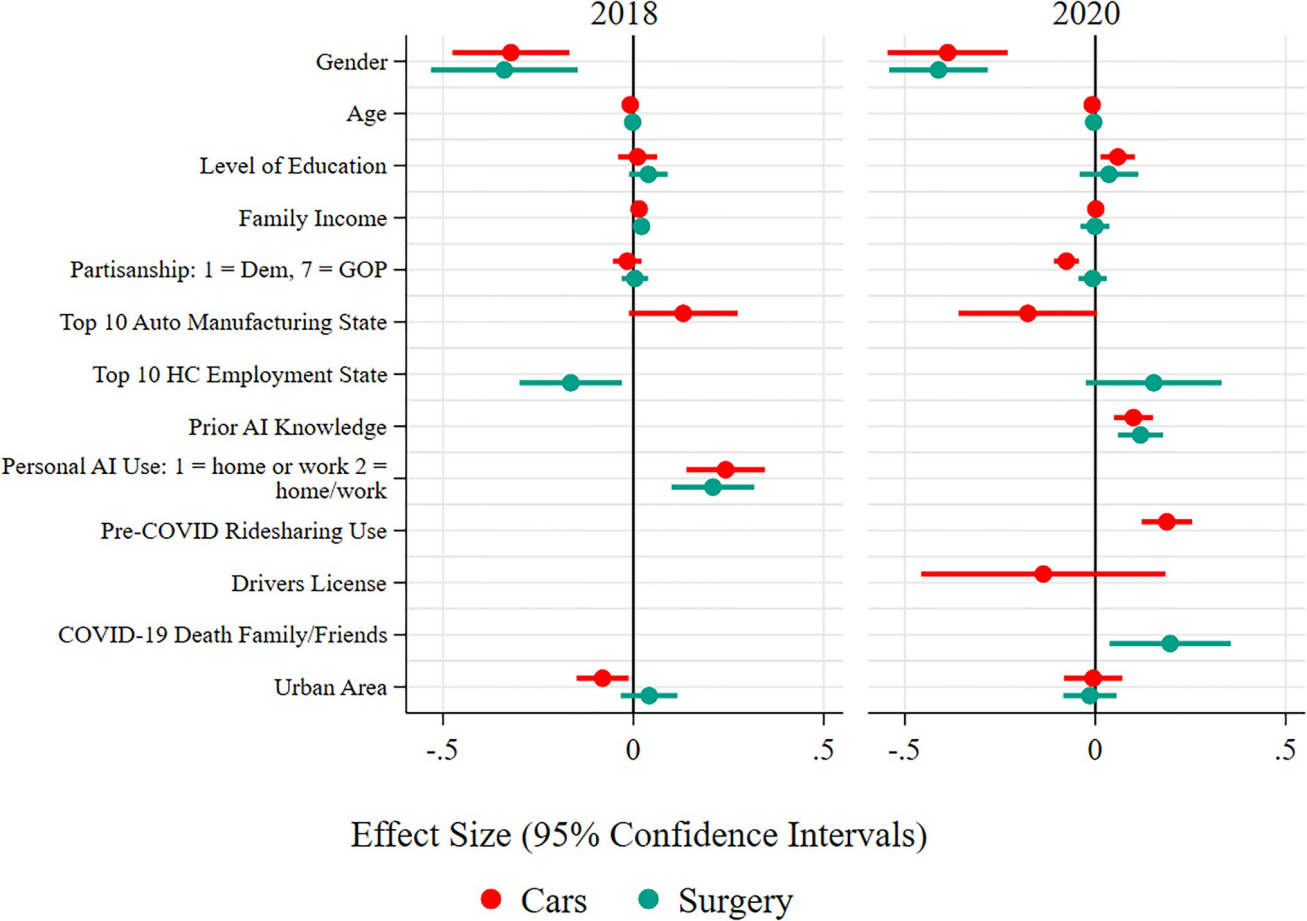
OLS regression analysis of support for vehicles and surgery, 2018 and 2020.

But can we attribute this effect to knowing someone who died from COVID-19? To assess how strong this experience might be for support for autonomous surgery, we turn to regression analysis, estimating another OLS regression model like the one for AI-enabled Autonomous Vehicles above. Support for autonomous surgery is the dependent variable and knowing someone who died from COVID-19 is an independent variable, along with potential confounders such as education, income, partisanship, and self-reported prior experience with AI. We graphically display the coefficients and 95% confidence intervals in Fig 4. These results show the statistically significant and substantively important role that knowing someone who died from COVID-19 plays even when controlling for confounders. Those that knew someone who died from COVID-19 are 19% more likely to support autonomous surgery than those that did not, controlling for a wide range of demographic and other variables, including income and education. The correlation is statistically significant at the 0.05 level. As with autonomous vehicles, the regression table is available in S1 and S2 Tables.

What else drives support for autonomous surgery? The OLS regression results graphically displayed in Fig 4 more broadly help illustrate key drivers of attitudes about autonomous surgery in general. As with autonomous vehicles, in both surveys, self-reported prior experience with AI is correlated with greater support for autonomous surgery, while women are significantly less supportive. Unlike with autonomous vehicles, there is no effect from age. Being in a top 10 health care industry state made support for autonomous surgery less likely in 2018, but more likely in 2020. This is also consistent with hypothesis 3, potentially, because it illustrates the way respondents in more health care-dependent states became more favorable about autonomous surgery due to COVID-19. There is no statistically significant correlation between partisanship and support for autonomous surgery in either the 2018 or 2020 results.

### Autonomous cyber defense and autonomous weapon systems

**Fig 5:**
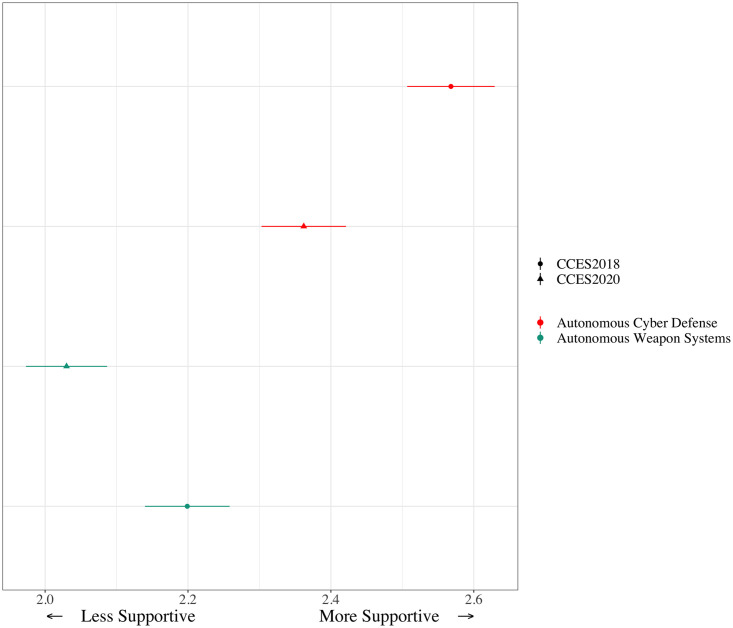
Support for autonomous cyber defense and weapons systems, 2018 and 2020.

We test hypotheses 4 and 5 by evaluating variation between 2018 and 2020 in public support for autonomous cyber defense and autonomous weapon systems. We begin again by looking at means and standard errors. Fig 5 shows decreases in support for autonomous cyber defense and weapons, in contrast to hypothesis 4. Support for autonomous weapons systems is lower in both 2018 and 2020 than support for any other AI application we test. This is consistent with prior research on public skepticism about algorithms making military decisions about the use of lethal force [21, 22]. The mean level of public support for autonomous weapon systems is 2.20 in 2018 and just 2.03 in 2020, decreasing from 40.7% somewhat or very supportive in 2018 to 31% in 2020. Even support for the use of AI in cyber defense decreases, from a mean of 2.56 in 2018 to 2.36 in 2020, a reduction from 57.6% somewhat or very supportive in 2018 to 47.3% in 2020.

**Fig 6:**
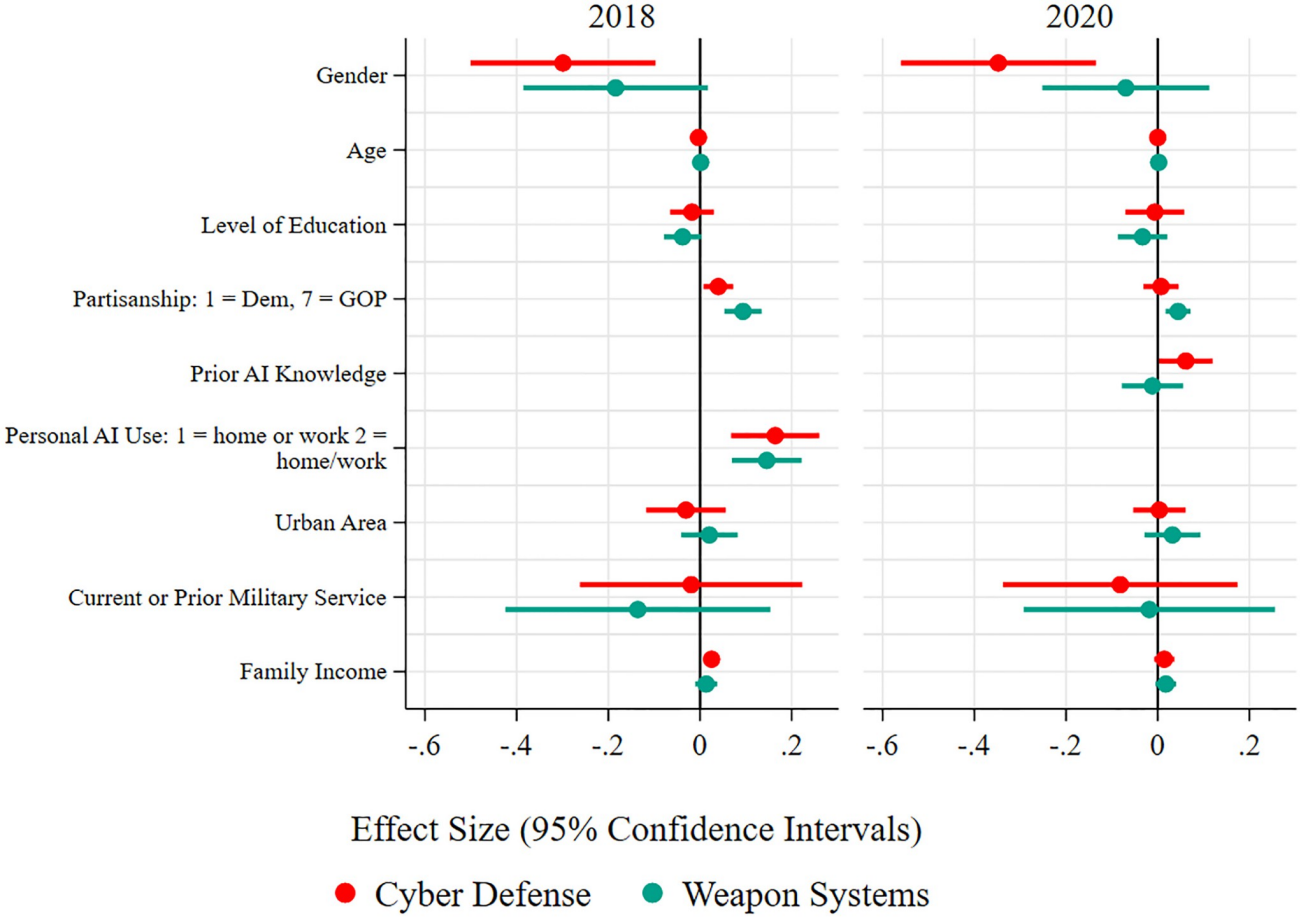
OLS regression analysis of support for autonomous cyber defense and weapons systems, 2018 and 2020.

What variables explain these patterns? As with the other AI-enabled autonomous systems, we estimate OLS regression models to test support for autonomous weapon systems and autonomous cyber defense in 2018 and 2020 with standard demographic and political variables, and self-reported prior experience with AI. The dependent variable is support for autonomous weapon systems or AI-enabled cyber defenses, and the independent variables are the independent variables described in the methodology section. The results are consistent using an ordinal logit specification given the 1–4 distribution of the dependent variable. The regression results are graphically presented in Fig 6 below. The regression tables are available in S1 and S2 Tables.

As Fig 6 shows, self-reported AI knowledge positively explains support for autonomous cyber defense in both surveys, as it does for autonomous vehicles and surgery. However, self-reported experience with AI does not make support for autonomous weapon systems more likely in 2020, only in 2018. Women appear less likely to support autonomous cyber defense and autonomous weapon systems in 2018, but only less likely to support cyber defense in 2020. The non-significant finding for gender for autonomous weapon systems in 2020 may be due to the much lower level of support for autonomous weapon systems overall. White respondents are less likely to support autonomous weapon systems in 2020, but there were no effects for either autonomous cyber defense or autonomous weapon systems in 2018, and there is no hypothesized reason for this result. There is some evidence of a partisanship effect for support for AI-enabled autonomous systems with autonomous weapon systems. In 2018, self-identified Republicans are significantly more likely to support both autonomous weapon systems and autonomous cyber defenses, though, in 2020, Republicans were only more likely to support autonomous weapon systems.

## Conclusion

These results show that attitudes about AI-enabled autonomous systems remain mixed in the US public, despite the COVID-19 pandemic. The results suggest that the impact of COVID-19 on support and opposition to AI-enabled autonomous systems is more complicated than initially hypothesized. Though there are reasons to think that over time, and with COVID-19, there might be an increase in public support for AI-enabled autonomous systems, it is not consistent across technologies. In our study, we find declining support across most of our issue areas, except in the case of autonomous surgery among those who know someone who died of COVID-19.

One possible explanation for these findings is that, except for those with direct experience of loss, COVID-19 may have made many individuals more risk averse and cautious of technological solutions, despite making people more dependent on digital technologies than ever before. The results also suggest that people are less likely to support AI-enabled technology when applied directly to their life, and opposition to some AI-enabled military applications has only increased over time, with Republicans more likely to support those applications, on average, than Democrats. All of these findings suggest that the proliferation of these technologies is a complicated phenomenon and one in which even a pandemic could not generate uniform support for autonomous technologies.

There are also limitations to our findings, which can serve as an instigator for future work. First, we only survey US adult respondents. A more global sample would test whether these results are more generalizable. Second, we would have asked more questions about personal health care experiences, risk propensity, or related topics, but were limited in the 2020 survey based on what was in the 2018 survey. Third, future research could more directly integrate gender and partisanship into the hypotheses in ways that could build knowledge.

In general, our work gives insights into the adoption of four types of significant autonomous technologies in the light of a historic pandemic with a snapshot taken at the height of COVID-19 mitigation policies when fears and uncertainty were high. Since then, individuals’ attitudes about COVID-19 mitigation measures have become sticky political beliefs, and therefore support for autonomy as a COVID-risk mitigation measure may have significantly changed (in both directions). A third survey fielded after the COVID-19 pandemic recedes in public imagination would reveal the extent to which any pandemic-correlated attitude changes might be long-lasting.

## Supporting information

S1 Table AI-enabled autonomous system support regression analysis, 2018. (PDF)

S2 Table AI-enabled autonomous system support regression analysis, 2020. (PDF)

S1 Fig Correlation of key independent and control variables (2020). (TIF)

S2 Fig Correlation of key independent and control variables (2018). (TIF)

S1 File Survey text. (PDF)
