# Supplementary material for: COVID-19 and public support for autonomous technologies—Did the pandemic catalyze a world of robots?
Source: PLoS One. 2022 Sep 28;17(9):e0273941. doi: 10.1371/journal.pone.0273941 (PMC9518891; doi:10.1371/journal.pone.0273941)
Supplement: S1 Table — (PDF) [file pone.0273941.s001.pdf]

S1 Table.

S1 Table: AI-Enabled Autonomous System Support Regression Analysis, 2018.

	(1) Cars b/SE	(2) Surgery b/SE	(3) Cyber Defense b/SE	(4) Weapon Systems b/SE
Gender	-0.321***	-0.339***	-0.299***	-0.184*
	(0.076)	(0.096)	(0.100)	(0.100)
Age	-0.008***	-0.002	-0.003	0.002
	(0.002)	(0.002)	(0.003)	(0.002)
Level of Education	0.011	0.039	-0.018	-0.038*
	(0.025)	(0.025)	(0.024)	(0.020)
Family Income	0.015	0.022**	0.025***	0.013
	(0.010)	(0.010)	(0.008)	(0.012)
Partisanship: 1 = Dem, 7 = GOP	-0.016	0.004	0.040**	0.094***
	(0.019)	(0.017)	(0.016)	(0.020)
Top 10 Auto Manufacturing State	0.131*			
	(0.071)			
Top 10 HC Employment State		-0.164**		
		(0.067)		
Prior AI Knowledge	0.242***	0.209***	0.164***	0.146***
	(0.051)	(0.054)	(0.048)	(0.038)
Urban Area	-0.081**	0.041	-0.031	0.020
	(0.034)	(0.037)	(0.043)	(0.031)
Current or Prior Military Service			-0.019	-0.136
			(0.121)	(0.144)
Constant	2.856***	2.314***	2.577***	1.946***
	(0.125)	(0.206)	(0.235)	(0.151)
Observations	863	863	863	863
R^2	0.115	0.081	0.072	0.074
Log Likelihood	-1158.252	-1163.314	-1170.829	-1154.365
F	32.966	8.544	10.892	6.335

Notes: St*p<0.10; **p< 0.05; ***p<0.01.
