# Supplementary material for: COVID-19 and public support for autonomous technologies—Did the pandemic catalyze a world of robots?
Source: PLoS One. 2022 Sep 28;17(9):e0273941. doi: 10.1371/journal.pone.0273941 (PMC9518891; doi:10.1371/journal.pone.0273941)
Supplement: S2 Table — (PDF) [file pone.0273941.s002.pdf]

S2 Table.

S2 Table: AI-Enabled Autonomous System Support Regression Analysis, 2020.

	(1) Cars b/SE	(2) Surgery b/SE	(3) Cyber Defense b/SE	(4) Weapon Systems b/SE
Gender	-0.388***	-0.412***	-0.348***	-0.070
	(0.079)	(0.064)	(0.106)	(0.091)
Age	-0.008***	-0.004	-0.000	0.002
	(0.002)	(0.003)	(0.002)	(0.003)
Level of Education	0.059**	0.036	-0.006	-0.033
	(0.023)	(0.038)	(0.032)	(0.027)
Family Income	0.001	-0.001	0.014	0.018
	(0.009)	(0.019)	(0.011)	(0.011)
Partisanship: 1 = Dem, 7 = GOP	-0.076***	-0.007	0.007	0.044***
	(0.016)	(0.019)	(0.019)	(0.014)
Top 10 Auto Manufacturing State	-0.177*			
	(0.091)			
Top 10 HC Employment State		0.153*		
		(0.089)		
Prior AI Knowledge	0.100***	0.119***	0.061**	-0.011
	(0.026)	(0.030)	(0.029)	(0.033)
Pre-COVID Ridesharing Use	0.188***			
	(0.033)			
Drivers License	-0.136			
	(0.160)			
Urban Area	-0.006	-0.014	0.004	0.032
	(0.038)	(0.035)	(0.028)	(0.030)
Current or Prior Military Service			-0.082	-0.018
			(0.127)	(0.136)
COVID-19 Death Family/Friends		0.197**		
		(0.079)		
Constant	2.794***	2.730***	2.604***	2.221***
	(0.234)	(0.197)	(0.204)	(0.210)
Observations	818	874	874	874
R^2	0.200	0.114	0.058	0.064
Log Likelihood	-1057.748	-1134.013	-1165.284	-1158.325
F	20.797	15.195	10.491	7.761

Notes: St*p<0.10; **p< 0.05; ***p<0.01.
