# Supplementary figures and images for: COVID-19 and public support for autonomous technologies—Did the pandemic catalyze a world of robots?
Source: PLoS One. 2022 Sep 28;17(9):e0273941. doi: 10.1371/journal.pone.0273941 (PMC9518891; doi:10.1371/journal.pone.0273941)

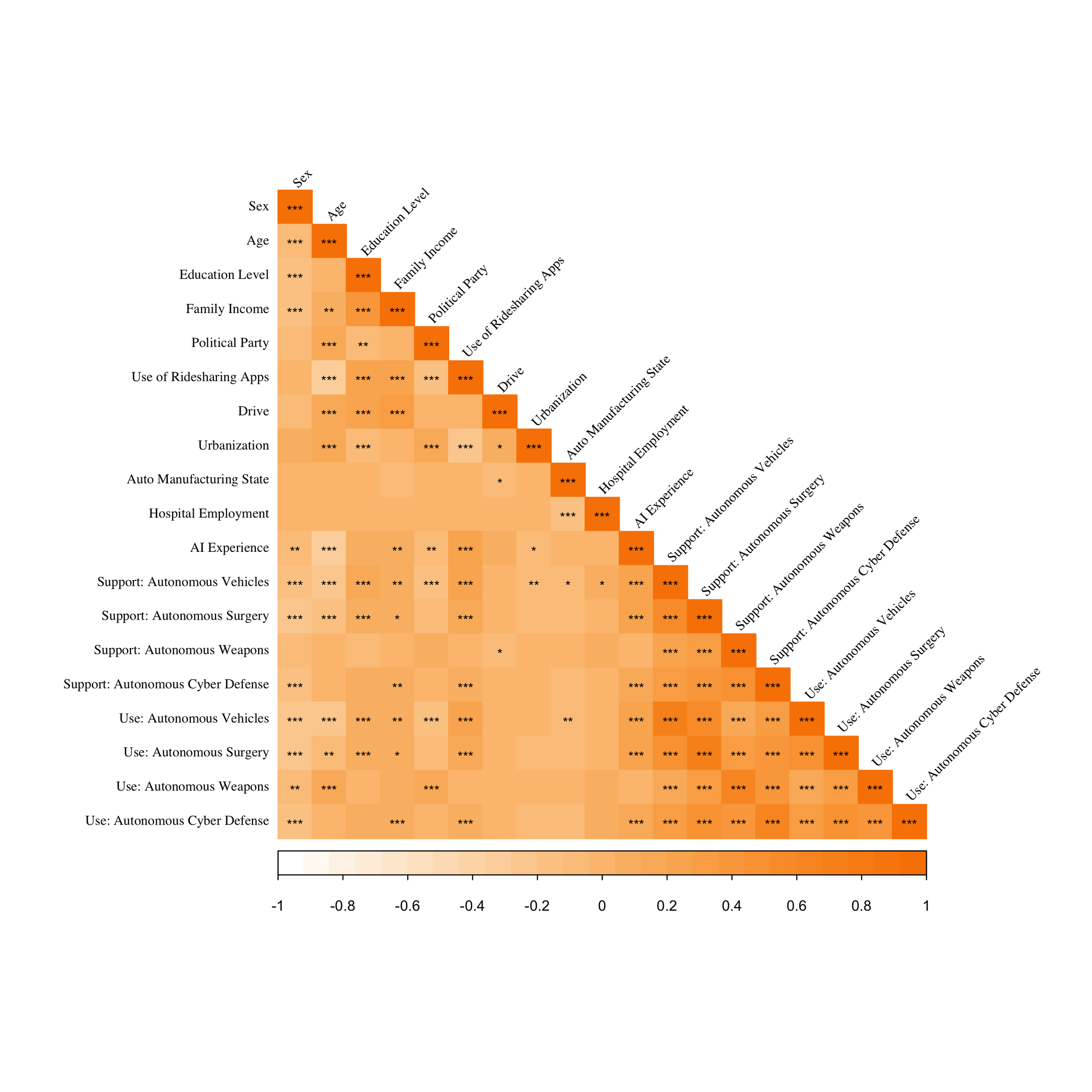

Supplement: S1 Fig — (TIF) [file pone.0273941.s003.tif]

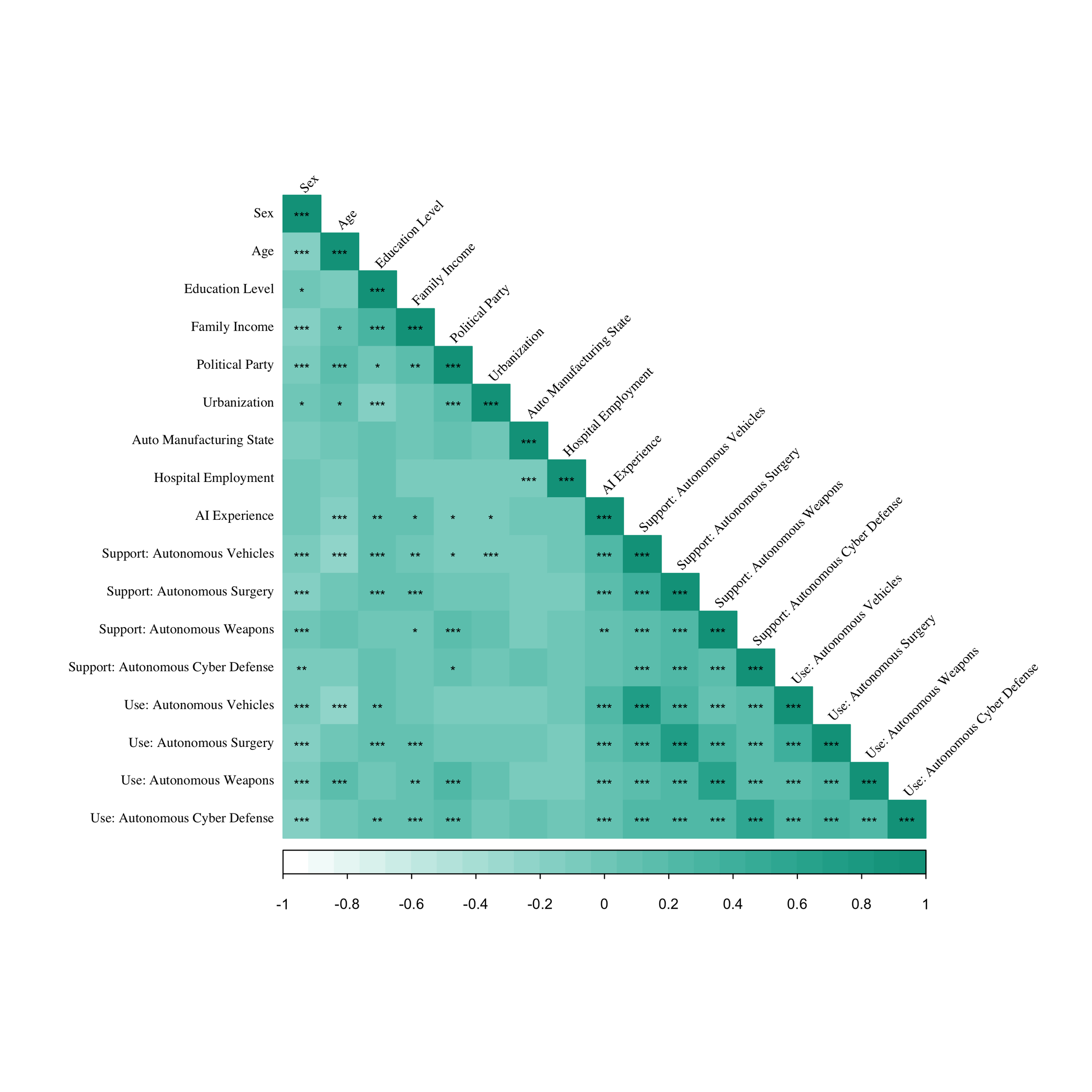

Supplement: S2 Fig — (TIF) [file pone.0273941.s004.tif]
