# Supplementary material for: COVID-19 and public support for autonomous technologies—Did the pandemic catalyze a world of robots?
Source: PLoS One. 2022 Sep 28;17(9):e0273941. doi: 10.1371/journal.pone.0273941 (PMC9518891; doi:10.1371/journal.pone.0273941)
Supplement: S1 File — (PDF) [file pone.0273941.s005.pdf]

S1 Survey Text.

Vehicles1 Prior to the COVID-19 pandemic, how frequently did you use ridesharing apps such as Lyft and Uber?

- Never
- A few times a year
- A few times a month
- A few times a week
- Almost every day or more
- I do not know what ridesharing apps are

Vehicles2 Imagine that automotive technology has advanced to the point where self-driving cars and trucks require little or no input from humans to operate. Would you support or oppose this development?

- Strongly support
- Somewhat support
- Somewhat oppose
- Strongly oppose

Vehicles3 How concerned are you about the safety of autonomous vehicles for those riding in them as well as for other vehicles, cyclists, and pedestrians?

- Not at all concerned
- Slightly concerned
- Very concerned
- Extremely concerned

Vehicles4 How likely would you be to ride in an autonomous vehicle?

- Very likely
- Somewhat likely
- Somewhat unlikely
- Very unlikely

Healthcare1 Imagine that medical technology has advanced to the point where surgical procedures will be able to be performed by autonomous systems (systems trained by an algorithm) with little to no input from humans. Would you support or oppose this development?

- Strongly support
- Somewhat support
- Somewhat oppose
- Strongly oppose

Healthcare2 How concerned are you about the safety of procedures for patients who undergo surgery conducted by autonomous systems?

- Not at all concerned
- Slightly concerned
- Very concerned
- Extremely concerned

Healthcare3 Would you support or oppose the use of artificial intelligence to make decisions about the allocation of limited health care resources?

- Strongly support
- Somewhat support
- Somewhat oppose
- Strongly oppose

Weapons1 Imagine that military technology has advanced to the point where autonomous weapon systems that require no input from humans after activation will be able to target and fire weapons. How supportive would you be of this development?

- Strongly support
- Somewhat support
- Somewhat oppose
- Strongly oppose

Weapons2 How concerned are you about the safety of autonomous weapon systems for the militaries that operate them?

- Not at all concerned
- Slightly concerned
- Very concerned
- Extremely concerned

Weapons3 How concerned are you about the safety of autonomous weapon systems for civilians?

- Not at all concerned
- Slightly concerned
- Very concerned
- Extremely concerned

Weapons4 How likely would you be to support the use of autonomous weapon systems to carry out a military mission of high importance to US national security?

- Very likely
- Somewhat likely
- Somewhat unlikely
- Very unlikely

Cyber1 Imagine that cyber defense technology has advanced to the point where automated responses to cyber attacks require no input from humans to operate after activation. How supportive would you be of this development?

- Strongly support
- Somewhat support
- Somewhat oppose
- Strongly oppose

Cyber2 How concerned are you about the safety of autonomous cyber defense for those systems utilizing the technology as well as critical infrastructure dependent on these systems?

- Not at all concerned
- Slightly concerned
- Very concerned
- Extremely concerned

Cyber3 How likely would you be to use autonomous cyber defense technology?

- Strongly support
- Somewhat support
- Somewhat oppose

- Strongly oppose

AIHome-Work (1 point for AI Index for each of home and work) Generally speaking, do you use artificial intelligence at work or at home?

- At home
- At work
- At work and at home
- Neither

AIMusic-Movies (1 point for AI Index if yes) Do you use artificial intelligence-based systems to select music or movies for your enjoyment (e.g. Pandora or Netflix)?

- Yes
- No

ML Knowledge A (1 point for AI Index if correct) Choose the option that is not correct regarding artificial intelligence,

- Artificial intelligence includes techniques that allow systems to learn without being explicitly programmed
- Machine learning is not a type of AI
- Artificial intelligence is often categorized into two types: general Artificial intelligence and narrow Artificial intelligence
- Artificial intelligence is a software, machine, or computer that researchers think could eventually emulate the human mind

ML Knowledge B (1 point for AI Index if correct) Which of the following is NOT an example of supervised learning?

- Principal Component Analysis
- Decision Tree
- Linear Regression
- Naive Bayesian
